# George Huntington (1850–1916)

**DOI:** 10.1007/s00415-018-8860-5

**Published:** 2018-04-30

**Authors:** Michał K. Owecki, Anita Magowska

**Affiliations:** Department of History and Philosophy of Medical Sciences, Poznań University of Medical Sciences, ul. Przybyszewskiego 37A, Poznań, Poland

*To the memory of Magdalena P. and all my patients with Huntington’s disease that I was unable to help as I wished*.

The nineteenth century was a time when specializations evolved from general medicine. General physicians, out of necessity dealing with the majority of their patients’ health problems, sometimes made important observations relevant to particular emerging specializations. For instance, a single article might unintentionally become an unquestioned and significant contribution to the development of a specific field of medicine. A good example in this context is George Huntington, the American family doctor after whom Huntington’s disease (or Huntington’s chorea) is named.

**Fig. 1:**
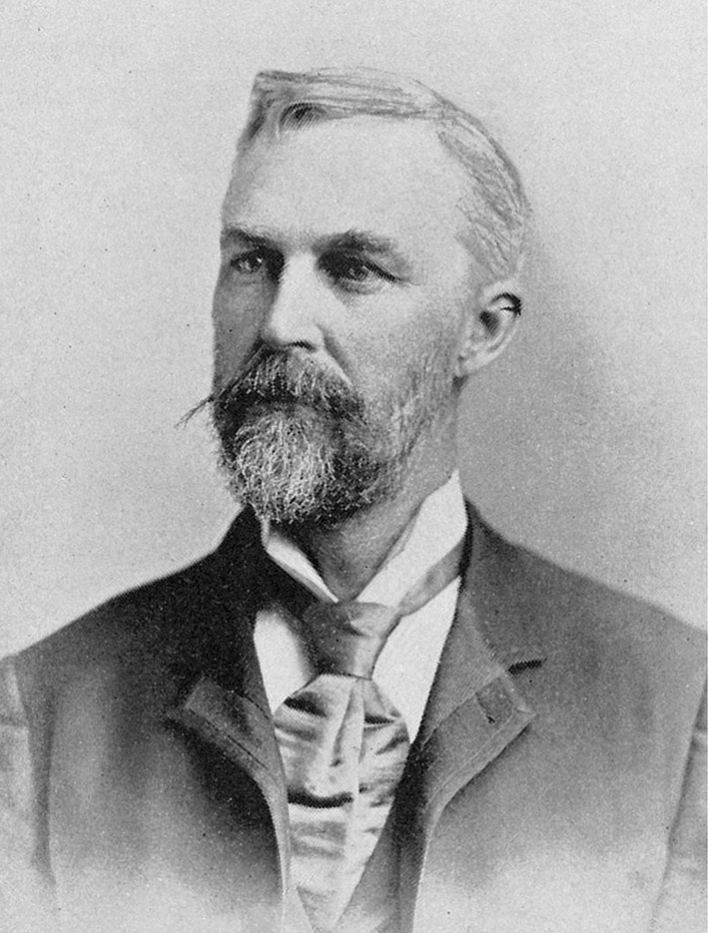
George Huntington (public domain)

George Huntington (Fig. 1) was born on April 9, 1850, in East Hampton on Long Island, New York, to a family with a rich medical tradition. He represented the third generation of physicians: his father, George Lee Huntington (1811–1881), had continued the medical practice opened by his own father, Abel Huntington (1777–1858). George Junior graduated from the Clinton Academy in his native town and, in 1868, began his medical studies at the College of Physicians and Surgeons at Columbia University in New York. In 1871, he qualified in medicine, with an inaugural dissertation on opium, at the age of 21. Shortly afterwards, he moved to Pomeroy in Ohio, where he began work as a family doctor [1].

On February 15, 1872, young Huntington traveled to the neighboring town of Middleport, Ohio. As it would turn out, the destination of that trip of a few miles was much farther: to a permanent place in the history of medicine. In front of the local medical society at the Meigs and Mason Academy of Medicine, a 22-year-old family doctor from a small town in the East of the United States, only 1 year after completing his medical graduation, with little clinical experience, gave a speech “On chorea”. Huntington must have been surprised by the significant difference he had noticed when comparing Sydenham’s chorea during his studies with those cases of chorea seen at his father’s practice. This probably explains why he chose to speak on chorea as a kind of welcome introduction to his new medical colleagues [2]. In his lecture, Huntington presented the clinical characteristics of chorea but, paradoxically, he dedicated the majority of the speech to discussing the symptomatology of Sydenham’s disease. Only in the final paragraphs did he specify the clinical features of the type of chorea later named after him: its hereditary nature, the onset occurring at “adult or middle life”, the predisposition to developing psychiatric symptoms, and a tendency to suicide. He also pointed out the steady progression of the disease, without any remission. At that time, he considered this form of chorea to be exclusive to Long Island and restricted “fortunately [to] a few families”; this was probably the reason he finished his lecture claiming that he only presented a “medical curiosity” with little practical significance. Time has shown how wrong he was in this opinion. The applause that followed the lecture prompted him to send his manuscript to *The Medical and Surgical Reporter* in Philadelphia, where it was printed only 8 weeks later, on April 13, 1872 [3].

This scientific report was noticed and published in the same year in the form of an abstract in the German medical press by Adolf Kussmaul (1822–1902) and Carl Nothnagel (1841–1905) in *Jahresbericht* or the Yearbook of Important Medical Writing for the Year 1872. Thus, was George Huntington’s name introduced into European medical literature [4]. The increasing recognition of the disease and, hence, growing interest in it contributed to further medical case reports that presented its clinical symptomatology consistent with Huntington’s own perception. In fact, the accurate and comprehensive characterization of chorea presented by George Huntington has become one of the classical descriptions of neurological diseases, and the reason the disease, in a period of eponyms, was named after this modest American physician. Several years later, in 1887, the designation “Huntington’s chorea” was coined by Huber [5]. But no man is an island. George Huntington had no patients of his own with the disease and his unquestioned success would probably have been impossible if he had not had the patients’ files he had inherited from his father and grandfather. He never denied that valuable heritage. Both his ancestors recorded their observations, describing successive generations of patients entrusted to their care. George Huntington scrupulously analyzed those notes, keeping in his memory the gradual mental and motor deterioration of the patients he saw in his childhood as he accompanied his father on visits to the sick in East Hampton. That unsettling view of the sick must have made a lasting impression on the child’s mind, as Huntington returned to the subject at the first opportunity. Also worth noting is Huntington’s father’s help and involvement in his son’s manuscript. His father introduced comments and suggestions that were then included in the final version of the article [2, 6].

Huntington most probably could not have known that the first comprehensive description of the disease later named after him had appeared in medical documents several years earlier. In 1860, Johan Christian Lund (1830–1906), a Norwegian physician from Setesdal, reported an inheritable and progressive form of chorea, although he called it St Vitus’ Dance; the latter, a condition distinct from Huntington’s, is now known as Sydenham’s chorea after the English physician Thomas Sydenham (1624–1689). In his paper on the health of the populations in the region and town in which he lived and worked, Lund had used the term “rykkja” or “Setesdalsrykkja” after the place name. However, Lund’s work was a kind of official statistical medical report for the year 1859 and was not translated into English at the time, which effectively limited its spread and visibility in the contemporary medical literature. The contribution of this Scandinavian doctor has largely been overlooked [7].

Despite such a brilliant and promising beginning to his career, Huntington did not choose a path of academic development or university research. Like his father and grandfather, he decided to live the ordinary life of a general practitioner, outside academic struggle. In 1874, he married Mary Elizabeth Hackard from Pomeroy, with whom he returned to his hometown of East Hampton. There, he fruitlessly tried to run a medical practice. After a few months, the young couple moved to LaGrangeville, New York, in the fall of the same year, where Huntington successfully worked as a rural general physician until 1901 [2, 8].

Huntington led a happy and peaceful family life in the countryside, sharing his time between professional duties, his relatives and his hobbies. He liked to draw wildlife, hunt and go fishing. Huntington loved music and often used to play the flute to his wife’s piano accompaniment. He paid a lot of attention to raising their five children, encouraging them to learn and work hard, offering them advice and sharing his knowledge. He undoubtedly adored his children and was loved by them. The second son, Edwin Horton, after some hesitation and to Huntington’s great joy, also chose a medical career, thus becoming the fourth successive generation of doctors in the family [8].

George Huntington suffered from severe attacks of asthma and, because of health problems, moved to Asheville in North Carolina in 1901, from where, after a 2-year convalescence, he returned to New York State in 1903 to the town of Hopewell Junction, neighboring LaGrangeville. He took up his practice again and for some time served as a visiting physician to the Matteawan General Hospital and as a health officer in Fishkill. His friendly temperament, mild manners and sense of humor, combined with a high level of professionalism, meant that Huntington never complained about a lack of patients. Some of his patients were very poor local farmers, but he never refused to treat people who could not afford his medical services. Doctor Huntington continued his work until 1915, the year he retired due to deteriorating health [1, 8].

Throughout his entire professional life, George Huntington vigorously participated in the activities of several local medical associations, acting for a period as vice-president (in 1887) and then elected president (in 1888) of the Dutchess County Medical Society. His contribution to neuroscience was recognized during his lifetime and, in 1898, he became an honorary member of the Brooklyn Society of Neurology [8, 9]. Thirty-six years after his first presentation, a special edited edition of “Neurographs” devoted to the life and work of George Huntington and chorea was produced in 1908 [1]. The following year, on December 7, 1909, Huntington gave a public lecture at the invitation of the New York Neurological Society. In his speech, published in 1910, he recollected the history of the description of the disease, reaching for personal threads from his childhood [6].

The above events must have been a great distinction for Huntington and, at the same time, the crowning of his professional career. Moreover, his contribution to neurology placed the name of this rural general practitioner next to prominent clinicians also dealing with extrapyramidal system diseases, such as James Parkinson (1755–1824), Gilles de la Tourette (1857–1904), and Henri Meige (1866–1940).

George Huntington died from pneumonia on March 3, 1916 at the age of 66, at the home of his son Edwin in Cairo, New York [10].
